# The Impact of Competitive Swimming on Menstrual Cycle Disorders and Subsequent Sports Injuries as Related to the Female Athlete Triad and on Premenstrual Syndrome Symptoms

**DOI:** 10.3390/ijerph192315854

**Published:** 2022-11-28

**Authors:** Joanna Witkoś, Grzegorz Błażejewski, Magdalena Hagner-Derengowska, Kamila Makulec

**Affiliations:** 1 Faculty of Medicine and Health Science, Andrzej Frycz Modrzewski Krakow University, G. Herlinga-Grudzinskiego Street 1, 30-705 Kraków, Poland; 2 Department of Physical Culture, Faculty of Earth Sciences and Spatial Management, Nicolaus Copernicus University, Lwowska Street 1, 87-100 Toruń, Poland

**Keywords:** swimming, women, female athlete triad, menstrual cycle disorders, low energy availability, premenstrual syndrome, injuries

## Abstract

Background: An athlete’s menstrual cycle may be seriously disturbed when she undertakes a physical activity that exceeds the body’s adaptive capacity and/or applies dietary restrictions. The main aim of this study was to assess the impact of swimming training undertaken by participants of sport clubs on disorders of the menstrual cycle. Methods: The study involved 64 female athletes. The questionnaire utilized in this study was composed by the authors, however some of the questions were based on Low Energy Availability in Females Questionnaire (LEAF-Q). Results: 31.26% of swimmers reported that the menstrual cycle was stopped for more than 3 months, of which 21.88% had a menstrual absence for more than 6 months and 9.38% between 3 months and 6 months. Years of training were a positive predictor of the ‘more profuse bleeding’. There was a negative correlation between the disorders of the menstrual cycle, the body weight of the female participants (*p <* 0.05) and the body mass index (*p <* 0.01). It was found that with the severity of the degree of disorder in the menstrual cycle, the number of injuries among the surveyed swimmers increased (*p <* 0.05). Conclusions: The correct body weight of the participants was a positive predictor of the absence of the menstrual cycle disorders among the majority of women practicing swimming. Disorders in the menstrual cycle occurring in a certain percentage of the swimmers positively correlated with the number of injuries recorded among these swimmers. Swimming has been shown to alleviate some of the premenstrual symptoms.

## 1. Introduction

Swimming is one of the most popular sports in the world and the second largest sport at the Olympics based on the number of athletes competing in pool events. In 2012, the Olympic Games were watched by over 219 million people, and swimming was one of the most often watched sports competitions. The discussed sport discipline is based on the ability to move the body in water in a harmonious and coordinated way by performing specific movements of the upper and lower limbs [1,2,3]. Swimming is unique because it takes place in an aquatic environment and it requires multiple abilities, from reaction speed to proper joint and, what is more, in this physical activity there is a necessity for more energy than in sports performed in a terrestrial environment, due to the need to overcome the hydrodynamic water resistance [4,5].

The discussed physical activity can be done in a variety of styles and distances, including confined water (swimming pools) or open water (seas, lakes) [4]. Depending on the position of the body in water, the nature of the propulsion movements of both the lower and upper limbs, the following swimming techniques are distinguished: breaststroke, backstroke, freestyle (crawl), butterfly style and a combination of the above techniques in alternating style competitions. In swimming competitions, participants usually swim from 50 m to 200 m, individual medley is held over 200 m and 400 m, while in open water swimming, the champions most often compete at distances of 3 km. Swimming, in addition to the individual discipline, is also part of multisport races, such as triathlons, and there swimming distances depend on the type of triathlon and can total, for instance, 1.5 km, 1.9 km, or 3.8 km in Ironmen competitions. In contrast, at the Olympics swimming typically ranges from 50 m to 1.5 km and lasts from less than 20 s to more than 15 min [5].

Women’s participation in swimming competitions, as in all other sport disciplines, has become commonplace. Among young women undertaking training at the professional level, the commonly used training programs are based on frequent and long training sessions, which can have a real impact on disorders of the menstrual cycle, one of the most important physiological processes in a woman’s organism [6,7,8]. A menstrual cycle within norm is a clear indication of a woman’s health, fertility and reproductive ability. Most physiological processes in the female reproductive system involve complex interactions between various tissues, hormones, and organs, including the hypothalamus, pituitary gland, ovaries, and uterus [9]. Unfortunately, even a normal menstrual cycle can be seriously disturbed if a woman undertakes physical activity that exceeds the body’s adaptive capacity and/or applies dietary restrictions [10].

Eating disorders, menstrual disorders and decreased bone mineral density (BMD)—which often leads to injuries, are the three components of the syndrome called Female Athlete Triad—FAT, to which women who are involved in sports that require a slim body are particularly vulnerable [6,7,8,11,12]. Therefore, female swimmers, similarly to gymnasts, skaters, and dancers, may be at risk of occurring FAT because these sports activities are characterized by performance in skimpy outfits and a desire to have a slim body shape and sometimes unrealistically low body weight [13,14,15]. However, by contrast to the above information, attention should be paid to the fact that there exist many theories trying to explain why female swimmers appear to be relatively protected against the development of menstrual disorders and the triad, compared to other female sportswomen [16].

However, FAT syndrome and its components described above constitute only a small part of the complex of symptoms associated with low energy availability (LEA) much more widely discussed in the academic literature, called Relative Energy Deficiency in Sport (RED-S) [17,18,19]. Both RED-S and FAT are a consequence of dietary restrictions, including both those undertaken consciously and those resulting from the lack of knowledge about the adequate supply of calories during physical activity and sports training [11,12,17,18,19]. RED-S is a set of symptoms occurring when the amount of energy consumed with food is insufficient in relation to the current needs of the organism. Insufficient energy intake disturbs the organism homeostasis and this effect can be exacerbated when a person engages in a physical activity at the level of regular sports training. LEA leads to many very serious, negative health effects affecting all organisms systems, including the female reproductive system [20,21,22,23]. In order to maintain proper functions of the organism, the exercising woman should maintain the energy consumption at the level of 45 kcal/kg fat-free mass/day, as providing energy below 30 kcal/kg fat-free mass/day inhibits, e.g., reproductive functions [12]. It is necessary to pay special attention to the fact, that the amount of calories supplied to the athlete’s body during strenuous sports training should be high enough to prevent RED-S and FAT. Overall, estimates show that up to 50% of exercising women have gentle menstrual disorders, and 33% have no menstrual bleeding at all [13]. The negative energy balance present in the organism is unable to support and maintain its important functions, including: homeostasis, growth, reproduction and, therefore, neuroendocrine systems adapt to negative conditions by “switching the body” to the functions that are the most significant at a given moment, thus the survival of the organism. It creates a situation where the right amount of energy is not supplied to these tissues and organs that are not directly related to the survival of the organism. The mechanisms of the “energy saving mode” come from the evolutionary legacy aimed at securing the survival and function of those tissues of the body that are necessary to maintain life in times of hunger [20,21,22,23]. Interestingly, as a result of these adaptations, the energy balance and consequently body weight may remain at a certain “task point” representing an apparent state of homeostasis that masks the actual lack of energy available for optimal physiological functions [20,21,22,23].

The relationship between menstrual disorders and sports injuries is apparent [24,25,26,27]. A study Mallinson et al. [25] found that female athletes who have one triad component have a risk of developing stress fractures about three times higher than athletes without triad components. For women with two or three components of triad, the risk of stress fractures is up to five times higher than for athletes without them. Studies [25,26,27] have also shown that the greater the number of triad symptoms, the greater the risk of injuries associated with bone stress and low BMD.

Premenstrual syndrome (PMS) is a set of physical, behavioral, and psychological symptoms that occur among most women of childbearing age. Symptoms appear in the late luteal phase of the menstrual cycle and disappear about 2–4 days after the onset of bleeding [28]. Numerous international institutions and societies recommend aerobic training as an aid in relieving PMS symptoms, these include among others: The American College of Sports Medicine (ACSM), The American College of Obstetricians and Gynecologists [29] the Royal College of Obstetricians and Gynaecologists (RCOG), and the International Society for Premenstrual Disorders (ISPMD) [30]. Recommendations are mainly related to the fact that water reduces the gravitational pressure on the joints and muscles. The pressure of water improves venous return. It was found that nerves present in human skin react to the surrounding water environment by transferring information to deeper parts of the body, thus stimulating the immune system, regulating the secretion of stress hormones, improving blood circulation, decreasing pain sensitivity, improving brain circulation and impact on the hypothlamo-pituitary-adrenal axis. Water massage also soothes the nervous system, contributing to the feeling of relaxation [31].

In relation to the information presented above, the aim of this study was to assess the impact of swimming training undertaken by participants of sport clubs on disorders of the menstrual cycle. The study also determined whether menstrual cycle disorders occurring in female swimmers overlap with the occurrence of sports injuries. In addition, using the opportunity to study a group of swimming athletes, the impact of swimming training on the occurrence and the possible alleviation of the premenstrual symptoms associated with sport were assessed.

## 2. Materials and Methods

### 2.1. Participants

The study initially involved 96 adolescent female athletes, regularly practicing swimming. Subsequently, 32 women, who used contraceptives or any medications that could affect the woman’s menstrual cycle, were excluded from this group. Therefore, the final statistical analysis included 64 female athletes meeting the inclusion criteria for the study. The mean age of the swimmers included in the analysis was 24.69 ± standard deviation 2.15 years. The average body weight of the competitors was 64.16 ± 9.39 kg, height 171.88 ± 5.82 cm, and the body mass index (BMI) was 21.67 ± 2.62. Women represented sports clubs located in the city of Krakow. The criteria for inclusion in the study were the occurrence of menarche and undertaking swimming training at the professional level. The criteria for exclusion from the study were the use of hormonal contraception, declaration of amenorrhea resulting from other known factors not related to physical activity, e.g., pregnancy, polycystic ovary syndrome, hysterectomy. The lack of a correctly completed questionnaire also resulted in exclusion from the research.

Women trained on average from 11.69 ± 3.69 years. The duration of a single training session was 1.72 ± 0.37 h, and the number of training sessions per week was 6, which translated into an average of 12.77 ± 6.66 h of training per week. The main swimming style undertaken by the swimmers was the crawl, where 54.69% of women trained it, followed by breaststroke style (29.69%) and backstroke (15.63%). Most women, which is 43.75%, swam 5 to 7 km during a single training session, 39.06% of the female competitors swam 3 to 5 km, 14.06%—less than 3 km, and only two swimmers covered a distance of more than 7 km during one training session.

### 2.2. Questionnaire

The questionnaire utilized in this study was composed by the authors, however, in order to ask swimmers precise and properly formulated questions regarding the issues of this research and in order to receive answers that would be the most valuable and authoritative, part of the questions were based on a validated tool that assesses early symptoms related to energy deficiency in women related to FAT and RED-S, namely Low Energy Availability in Females Questionnaire (LEAF -Q). The questions about premenstrual symptoms have been added. Additionally, Rate of Perceived Exertion scale (RPE scale) was used for the study [32]. This tool was employed because it seemed interesting to compare the subjective assessment of the intensity level and the general perception of physical effort undertaken by the swimmers during training, in relation to the studied variables. The scale presented to the participants ranged from 6 to 20, where 6 meant “no effort at all” and 20 “maximal effort”. The swimmers were asked to choose a numerical value that best described the level of physical exertion experienced by the athlete during training. The sportswoman was asked to consider all subjective impressions and sensations regarding both physical stress and fatigue. The Numeric Rating Scale (NRS) was used to determine the subjective assessment of pain intensity experienced during menstrual bleeding, where 0 means no pain and 10 means unbearable pain.

### 2.3. Statistical Analyses

Statistical analyses were performed for the three statistical tests applied: chi-square, Spearman correlations, and logistic regression, using the SPSS 27 software program (Version 27.0, IBM Corp., Armonk, NY, USA). They were performed by means of G*Power software [33,34]. The achieved power in the case of the chi-square test, for small, medium, and large effects (phi = 0.1; 0.3, 0.5), respectively, was: 13%, 67%, and 98%. For Spearman correlations (rho = 0.1, 0.3, and 0.5; two-tailed tests): 12%, 70%, and 99%. For logistic regression (odds ratios 1.3, 3.0, and 5.0; proportion of participants on dependent variable was assumed as 0.2): 7%, 49%, and 83%. The achieved power was satisfactory in the case of large effects, but not medium or small ones.

## 3. Results

The athletes declared that the level of subjectively perceived physical effort related to the undertaken physical activity, i.e., swimming training, measured on the RPE scale, was 15.30 ± 2.16. Statistical analysis showed no significance between subjectively perceived physical exertion and disorders of the menstrual cycle, age of menarche appearance and pain experienced during menstrual bleeding. Studies have shown that all swimmers had their first menstrual period naturally. Among the vast majority of the women (70.31%), menarche appeared between the ages of 12 and 14, in 18.75% at or below the age of 11, and among 10.94%, only at the age of 15 or later. On average, the menarche among the female participants was 12.63 ± 1.50 years of age.

Disorders of the menstrual cycle, understood as the lack of menstrual bleeding, which did not occur after a period of regular bleeding, never occurred among 31.25% of the swimmers. To the question: “*are your periods regular*?” (Every 28th to 34th day) the majority of swimmers (70.31%) replied “*Yes, most of the time*”. However, when asked “*Have your periods ever stopped for 3 consecutive months or longer (besides pregnancy)*?” a total of 31.26% of the swimmers answered “*yes*”, of which 21.88% experienced a menstrual absence for more than 6 months, and 9.38% between 3 and 6 months. No bleeding lasting less than 3 months was reported among 37.50% of female athletes. There was a negative correlation between the disorders of the menstrual cycle, the body weight of the female athletes (*p <* 0.05) and the body mass index (*p <* 0.01). The higher the body weight of the athletes and the higher the BMI, the less frequent the menstrual cycle disorders among the swimmers. There was also a negative correlation between the athlete’s age and disorders of the menstrual cycle, but it was not statistically significant. What is more, a negative correlation was also established between the number of years of swimming training and the number of trainings per week, and the disorders in the menstrual cycle. However, this correlation did not show statistical significance (Table 1).

Women with menstrual disorders were asked to give a probable, subjectively considered reason for the situation. A significant percentage of swimmers (75.00%) indicated that too high intensity of the undertaken swimming training was the possible cause of the menstrual cycle disruption. Respectively, 20.45% of the women admitted to taking dietary restrictions and reducing body weight, while 2.27% of the participants indicated lack of sleep and dehydration.

By analyzing the swimming styles undertaken by the women and their possible impact on disorders of the menstrual cycle, statistical significance was found for disorders of the menstruation lasting less than 3 months and the swimming style undertaken, including the backstroke (Table 2).

On the other hand, no statistical correlation was found between the swimming style undertaken by the participants in the study and the pain experienced during menstruation and changes in the characteristics of the menstrual cycle (more or less heavy bleeding) (Table 3).

In order to obtain information on the possible impact of swimming training on the alleviation of pain associated with menstrual bleeding, the athletes were asked at the beginning whether they felt pain related to menstruation. The vast majority of swimmers (79.69%) answered “yes, but only at the beginning of bleeding“, subsequently, 18.75% declared that the menstruation was painful throughout the entire duration of the menstruation period, and only one woman (1.56%) said that she did not feel any pain during menstrual bleeding. Another question concerned the opinion on the effect of swimming training undertaken during menstruation on the increase or decrease in pain associated with menstrual period.

The participants were asked to mark the answer that best corresponded to the subjective experiences of the women. About half of the swimmers (52.38%) stated that their regular training reduces pain, 30.16% of the swimmers do not feel any influence of swimming training on any changes in pain feeling, and 11.11% believe that swimming training increases the pain accompanying menstruation. Four women, which accounted for only 6.35%, do not undertake training during menstruation. On the other hand, when analyzing the impact of swimming training undertaken during menstrual bleeding on the possible reduction of the pain level occurring and assessed by the respondents, appearing both at the beginning of menstrual bleeding and throughout its duration, no statistical correlation was found.

The swimmers were then asked to rate the overall level of pain they experienced during the menstrual bleeding on the NRS scale. The most female athletes (21.88%) declared that the pain they feel during menstruation corresponds to the number 8, successively: 17.19% indicated the number 9, 14.06% the number 5, 12.50% the number 7, and 10.94% the number 6. The numbers 2, 3, 4 were, respectively indicated by 7.81%, 4.69% and 6.25% of the women. No pain and pain at the level of 1 and 10 were indicated only by individual persons, which was only 1.56%. Subsequently, the swimmers indicated numbers that express the level of pain experienced when they undertake training during the period of menstrual bleeding. It was noted, that the numerical values for the level of pain experienced during swimming training in the period of menstrual bleeding changed, compared to the pain present without training. In particular, the differences concerned the numbers describing the intensity of pain at higher levels, i.e., above the number 5. For example, without taking up sports training, the number 8, which means relatively intensive pain, was indicated by 21.88% of women, while the same number was indicated by only 6.25% of female swimmers during menstrual bleeding. Number 9 also decreased from 17.19% without training to 9.38% during menstruation and training. The percentage of women choosing the number 7 was 9.38% with training vs. 12.50% without training. However, it is surprising to notice and record an increase in the percentage of women declaring the level of pain described by the number 6 from 10.94% without training to 17.19% during training, a clear increase was also noted for the number 3 from 4.69% without training to 15.63% during training and for the number 0 from 1.56% without training up to 6.25% during training. There were no noticeable differences for the remaining numbers on the NRS scale. Analyzing the impact of swimming training undertaken during menstrual bleeding on the possible reduction of the pain perception level present and assessed with a numerical scale by the participants in the study, a positive correlation of rho = 0.68 (*p <* 0.05) was found between the pain experienced by women during menstrual bleeding and the pain experienced during training at the time of menstruation.

Changes in the monthly cycle, e.g., its shortening or prolongation, can be caused by, e.g., changing the intensity of training. Therefore, the athletes were asked whether they felt changes in the characteristics of the monthly cycle with the increase in intensity, frequency or duration of training. The majority of women (65.63%) stated that their swimming training influences changes in the monthly cycle. Among the above-mentioned women, the most (38.10%) reported that bleeding lasts less days, i.e., it has been shorter, successively 35.71% said that the menstrual bleeding is more extensive, 21.43% said that the bleeding is less heavy, and 4.76% said that the bleeding lasts more days.

Correlation analysis showed, that with the increase in the number of years of swimming training among the female athletes, the frequency of heavy monthly bleeding increased (*p <* 0.05). There was also a negative correlation between the athlete’s age and the duration of monthly bleeding. The older the swimmer was, the less frequently the bleeding lasted for more days than the generally accepted norm of normal bleeding (*p <* 0.05). There was also a statistically significant relationship (*p <* 0.05) between BMI and reduction of heavy periods. The correlation was negative, which meant that the higher the BMI of a given athlete, the less frequent scanty bleeding (Table 4). Statistical analysis confirmed the significance that occurs between the change in training, which may be, for example, an increase in the intensity, or the frequency or duration of training, and the symptoms observed by the athletes regarding the monthly cycle. Bleeding was more profuse with rho = 0.40 (*p <* 0.01), or lasted less days with rho = 0.44 (*p <* 0.01). A change in training also led to scanty bleeding rho = 0.29 (*p <* 0.05), or amenorrhea (lack of the menstruation) rho = 0.31 (*p <* 0.05). Changes in training did not significantly correlate only with the possible extension of the monthly cycle among the surveyed swimmers.

Symptoms of premenstrual syndrome can be alleviated by taking up physical activity, therefore the most important symptoms characterizing this syndrome were referred to in the questions asked swimmers. Most of the surveyed women (89.06%) experienced PMS symptoms. Among the most common symptoms reported by female athletes were: pain in the lower abdomen, such a response was provided by 13.90% of the athletes, irritability (13.37%), pain in the lumbosacral region (10.70%), water retention in the body (10.16%), tearfulness/depression (9.89%), breast hypersensitivity (8.56%), fatigue (8.29%), diarrhea (6.69%), skin lesions (5.88%), headache/dizziness (5.08%), nausea/vomiting (4.81%), arthralgia (2.67%). Subsequently, the participants were asked to indicate the symptoms of PMS that, according to them, are alleviated by undertaking physical activity such as swimming training. The majority of women stated that swimming reduced lower abdominal pain (23.44%) and irritability (29.69%), successively swimmers indicated relief of pain in the lumbosacral region, such an answer was provided by 18.75% of participants, reduced tearfulness and depression—8.59%, oedema reduction (6.25%) and fatigue reduction (4.69% of swimmers). A positive effect on the reduction of pain and dizziness was indicated by 3.91% of the swimmers, only individual persons indicated reduction of other PMS symptoms. Statistical analysis showed a negative correlation of *p* < 0.05 between the number of years of swimming training and the sum of PMS symptoms alleviated by this type of physical activity.

Injuries in sport are inevitable, but they can be aggravated by disorders in the menstrual cycle, therefore the research also addressed the issue of injuries suffered by swimmers occurring during the last year. Although the injuries and the area of their occurrence reported by swimmers are listed below, as these were the data that were collected during the research, they were not subjected to extensive analysis, because describing injuries in swimming would significantly exceed the capacity of this manuscript. The athletes were asked two questions, which were formulated as presented in the LEAF questionnaire: “Have you had absences from your training, or participation in competitions during the last year due to injuries?”, “If yes, for how many days absence from training or participation in competition due to injuries have you had in the last year?” The vast majority of female athletes (79.69%) reported an injury in the last year while taking up their sports career. The number of injuries among the above-mentioned athletes was different: the most female swimmers (76.00%) had an injury once or twice in the last year. The occurrence of three or four injuries in the last year was observed by fewer women, that is only 22.00%, and five or more injuries by only one woman, which constitutes 2.00%. The most common injury among the swimmers was joint inflammation, such an answer was provided by 31.58% of the respondents, followed by muscle strain (27.37%), muscle tear or strain (15.79%), contusion (12.63%), meniscus tears (7.37%) and desmorrhexis or ligament rupture (5.26%). The participants were also asked which area of the body was the most common part of injury and 36.56% of women replied that it was the shoulder girdle, 27.96% indicated the knee joint, 6.45% the ankle joint and the area of the head and neck, 5.38% indicated the hip joint and muscles of the lower limbs and 4.30% the elbow joint, the remaining single persons indicated other areas of the body. Most often, the injuries occurred during the training sessions- such an answer was given by 62.75% of the participants, the main reason was training overload occurring in 33.33% of women, the least frequent injury occurred as a result of an accident in the swimming pool, such a situation occurred in 3.92% of swimmers. A break in training caused by an injury among the largest percentage of athletes, that is in 41.18% of women, lasted from 1 to 7 days, in 29.41% of swimmers from 8 to 14 days, in 23.53% of women 22 days and longer. Convalescence among 5.88% of swimmers lasted from 15 to 21 days. A positive correlation was demonstrated between the height, body weight and the number of years of swimming training and the frequency of injuries among the female athletes (*p* < 0.05) (Table 2). Analyzing the disorders of the menstrual cycle among the female athletes as compared to the number of injuries, a statistically significant correlation was found: rho = 0.26 (*p* < 0.05), showing that the number of injuries increased with the severity of the menstrual cycle disorder.

Multiple logistic regressions (Table 5) showed that years of training were a positive predictor of the ‘more profuse bleeding’. No significant predictors were detected in the case of ‘less profuse bleeding’, ‘bleeding lasts less than 3 days’, ‘amenorrhea’, and ‘injuries incidence’. Analyses were impossible in the case of ‘bleeding lasts more than 3 days’—there were only two participants with this symptom. Cox and Snell R-squares were comparable across analyses indicating that the explanative power of the predictors did not differ much across models.

## 4. Discussion

Physical activity requires providing sufficient amount of energy to the organism, so that the competitor’s body can use it not only to maintain its basic functions, but also to the processes that are related to sport [20,21,22,23]. In the case of swimming, also to overcome water resistance and to move in water environment with variable bone-joint-muscular involvement depending on different swimming styles [13]. Despite the many indisputable advantages of practicing sports, there is a risk of athletes taking dietary restrictions aimed at achieving low body weight necessary for achieving greater physical efficiency and better results in competitions [14]. Although the study did not evaluate eating habits (a diary of daily energy consumption) of the swimmers, but only menstrual cycle dysfunction as one of the elements of FAT, the knowledge about the relationship between eating disorders and low energy availability with disturbances of the menstrual cycle has been repeatedly demonstrated in the studies of other authors [10,11,12,13,14,15] and it turns out to be apparent.

The presented research assessed the occurrence of sport-related menstrual cycle disorders in women undertaking long-term swimming training at the competition level. The findings of the authors’ own research showed that most of the athletes had menstruation cycles within norm, which was presumably related to the correct body weight and BMI within the normal range. Only about 8% of women had a BMI just below the norm. Irregular menstrual cycles were reported by 29.69% of women, which is a result similar to the results of the authors assessing menstrual cycle disorders in various groups of non-training women. For instance, in the study by Aber [35] conducted among students of fourth and fifth classes of curative medicine faculty, that is women of a similar age as the examined swimmers, it was found that 20.54% had irregular menstrual cycle. Similar results were obtained by Deborah et al. [36], who investigated prevalence of menstrual irregularities in correlation with body fat among students in India where 23.3% students had menstrual irregularities. However, secondary amenorrhea observed in the study group of female swimmers, investigated generally, without analyzing the number of months without menstrual bleeding, was reported by as many as 68.75% of athletes, which is a significant result compared to the incidence of secondary amenorrhea in the general population who do not participate in sports, ranges from 2% to 5% [8,12]. The results have shown that the occurrence of secondary amenorrhea in the examined group of swimmers was close to the upper limit of occurrence of menstrual cycle disorders among athletes, which ranges from 6% to 79% [6,7,11].

The results of this research show that the older the swimmer was, the less frequent the disorders of the menstrual cycle. The authors of this study also showed that with the increase in the number of years of swimming training among the athletes, the incidence of heavy monthly bleeding increased, which was also confirmed by the results of multiple logistic regressions analysis. Any change in training intensity or frequency resulted in changes in menstrual bleeding that became more or less heavy, or even stopped completely. However, it was satisfactory that all the female athletes of the authors’ own research had a natural menarche, on average around the age of 13, which is a normal phenomenon. For example, a study Sambanis et al. [37] showed that elite artistic swimmers had a menarche delay of 0.6 years. Beals et al. [38] examined the prevalence of disordered eating, menstrual dysfunction, and low bone mineral density among US collegiate athletes participating in lean-build and non-lean build sports. It was found that menstrual dysfunction was significantly more prevalent among lean-build athletes.

An interesting study showing results that contradict the above-mentioned research by Ramsay et al. [16] evaluating synchronized swimmers who practice a particular type of water sport requiring a high level of aerobic and anaerobic fitness combined with grace and agility. It was found that the elite synchronous swimmers from the UK were not at risk of developing menstrual disorders and therefore, were unlikely to have triad-related reduced bone mineral density. However, it is suspected that training (mostly without load) was not very beneficial for gaining appropriate bone density of these athletes [16].

The introduction to this paper mentions theories that try to explain why female swimmers may be particularly protected against the development of FAT.First, submerging the body in cool water may allow swimmers to increase internal body temperature more than, for example, runners or dancers, which in turn may result in less disruption of the hypothalamus. Second, swimmers typically have relatively higher levels of body fat than other athletes. This is not a coincidence, as more body fat allows swimmers to float higher, thereby improving their performance in the sport. Additionally, as mentioned before, a higher level of adipose tissue protects against disorders of the menstrual cycle [16].

In sport, especially in exercise tests, the Borg rating of perceived exertion scale [32] is a frequently used measure of subjectively perceived physical effort. The level of felt strain can be an important indicator for coaches and athletes to assess training intensity and athlete’s endurance. This study showed that the average score of subjective feeling of physical effort measured with the RPE scale was 15.30 ± 2.16. This result indicates the fact that for most of the athletes the undertaken swimming exercises were heavy, however, the percentage of maximum heart rate (86–91% HRmax) and the percentage of maximum oxygen consumption (76–85% VO2max) in this range are within the zone of optimal training. Probably, due to the fact that the training was within the optimal limits—according to the above scale, it did not show a significant effect on the dysfunction of the menstrual cycle.

Despite many unquestionable advantages resulting from taking up physical activity such as swimming, this sport, when practiced professionally, contributes to contusions and injuries. The results of our research showed that the most common health problem reported by female swimmers was shoulder injuries, declared by 36.56% of athletes. In addition, research has confirmed that with the severity of the degree of disorder in the menstrual cycle, the number of injuries among the swimmers significantly increased, which is unquestionably related to FAT.

Swimming training is characterized by a relatively large volume, intensity and cyclical repetition of movement in the shoulder joint. Musculoskeletal overuse injuries, particularly shoulder injuries, nutrition deficits, and overtraining are most commonly observed among swimmers. A multitude of issues contribute to the “swimmer’s shoulder” from hypomibility, instability, lack of strength/muscle imbalance, overuse, change in volume, inadequate recovery to coaching oversigh [39,40,41,42]. Despite the above facts, injuries in sports, including swimming, may be reported more often in women who have FAT syndrome, including estrogen deficiency and reduced BMD. This situation occurs despite the fact that not all three elements of the triad are always present. For instance, in the studies of Schtscherbyna et al. [43] it was found that among 78 elite swimmers aged 11–19, one component of the triad was present in 47%, while two components were present in a much smaller proportion of 15.4% and 1.3%. The findings of the authors’ own research has shown a strong relationship between the occurrence of menstrual cycle disorders and the incidence of injuries among the swimmers, which is consistent with the literature describing the triad syndrome of sportswomen [6,7,8,12]. The study Mallinson et al. [25] aimed to demonstrate the relationship between the triad risk assessment score and the number of sports injuries. In the study group there were 3.4% of sportswomen with LEA and 5.2% of those with amenorrhea, with none of the athletes having all three elements of the triad or bone mineral density (BMD). However, among the several groups of athletes examined, swimmers were found to be at a relatively low risk of injury compared to athletes engaging in other types of sporting activity.

When discussing the general occurrence of shoulder injuries in sports such as swimming, in the study by Kerr et al. [44] it was found that the main factors of the occurrence of injuries in swimming was a large training volume and the repetitive nature of the movement. Sein at al. [45] assumed that shoulder pain among swimmers is common and its pathogenesis is uncertain. The authors found that 91% of swimmers aged 13–25 had a shoulder joint pain occurrence, 84% had a positive impingement sign and 69% of 52 swimmers had supraspinatus tendinopathy. It was found that supraspinatus tendinopathy is the major cause of shoulder pain among elite swimmers. Walker et al. [46] assessed the frequency of pain in the shoulder joint among 74 professional swimmers aged 11–27 (including 37 men and 37 women). Research has shown that 38% of athletes reported significant interfering shoulder pain in the last 12 months of training, and 23% significant shoulder injury lasting for at least 2 weeks. The related pain disrupted or prevented the athletes from training and competing, which clearly indicates the traumatic nature of swimming associated with the shoulder joint and the resulting limitations appearing in the course of a sports career.

The results of our research showed a negative correlation between the number of years of swimming training and the sum of PMS symptoms alleviated by this type of physical activity. The majority of female athletes stated that swimming reduced lower abdominal pain (23.44%) and irritability (29.69%), successively swimmers indicated relief of pain in the lumbosacral region, as well as, reduced tearfulness and depression. In the study by Khademi et al. [47] comparing prevalence of premenstrual syndrome in swimmer and non-swimmer students it was found that PMS occurred in 36.2% non-swimmers and 22.8% swimmers. The prevalence of PMS was found to be lower in swimmers. Comparison of PMS symptoms between swimmers and non-swimmers showed a statistical difference between symptoms such as: feeling more irritable (swimmers 25.7% vs. non-swimmers 40%), tend to eat more than usual or at irregular hours (respectively 22.9% vs. 34.3%), easily distracted (22.1% vs. 32.9%), restless behaviour noticeable by others (19.3% vs. 30.7%), physical symptoms (17.1% vs. 30.7%), change in mood without obvious reason (14.3% vs. 31.4%), significant swelling in breasts, ankles, and abdomen (12.9% vs. 26.4%), avoiding some social commitments (3.6% vs. 13.6%). In the study by Maged et al. [48], swimming has been shown to have a beneficial effect on most of the physical and psychological PMS symptoms. There were statistically significant differences between the group of swimmers and the control group with regard to the reduction of symptoms, e.g., such as: anxiety, depression, tension, mood changes, confusion, pain, headache, fatigue, breast tenderness. No significance was found for irritability, insomnia, edema, crying and food craving. Scientific research indicates a positive effect of swimming on the reduction of depression, anxiety, and stress, which is associated with the release of neurotransmitters, including secretion of β-endorphins. The positive effect of this physical activity as a form of aerobic exercise in relieving nervous tension is also attributed to an increase in the level of progesterone, which, through neurotransmitters modulated by sex steroids (like gamma-aminobutyric acid and serotonin), reduces stress and mental tension [49]. 

The syndrome of relative energy deficiency in sport is a clinical entity, the basic mechanism of which is the lack of sufficient amount of energy from food needed to support optimal health and physical performance of the organism [17,19]. Dietary restrictions, and thus inadequate energy intake in the diet combined with physical exercise, cause a negative energy balance in the athlete’s body, leading to serious multi-system health problems, and thus a decrease in the athlete’s training performance and improper regeneration of the organism [17]. Unfortunately, competitors often manipulate dietary intakes, in order to control weight and body composition [14]. The research [13,14,15] has shown, negative health consequences that do not occur immediately may turn out to be insufficient for an athlete to change their eating habits. The factor motivating an athlete to change the diet are only noticeable decrease in the body’s efficiency and a reduced response to training stimuli, and this already suggests significant health problems.

There is a clear need to spread knowledge about FAT and RED-S among athletes, and in particular among sportswomen, who should be aware that stopping the menstrual cycle while doing sport is not a comfortable situation allowing for undisturbed training, but the first symptom of energy disorders occurring in a woman’s organism leading to serious health consequences.

Limitations. The main limitation of this study, which is composition analysis was performed to determine the actual content of adipose tissue in the body of the examined women in relation to the lean body mass. The authors relied on the subjective statements of the athletes which may have made the study somewhat erroneous, but the data collected for analysis was mostly based on a questionnaire of unquestionable scientific value, which certainly contributed to the credibility of the obtained results. The main limitation of this study was the inability to perform long-term studies and to observe whether, for example, after stopping swimming training, all women had their menstruation cycle back to normal.

## 5. Conclusions

The research showed that the correct body weight of the athletes and BMI within the normal range were a positive predictor of the lack of disorders in the menstrual cycle among the majority of women practicing swimming. Disturbances in the normal menstruation cycle, occurring among a certain percentage of the swimmers, positively correlated with the number of injuries that were recorded among these athletes. Any change in the intensity, frequency or duration of training had an impact on the changes in menstrual bleeding, either becoming more or less heavy, or leading to amenorrhea. Years of training were a positive predictor of the ‘more profuse bleeding’. Swimming has been shown to improve some of the symptoms of PMS. Swimming styles taken as dominant among the examined women did not significantly change the characteristics of the menstrual cycle, but the backstroke significantly influenced the absence of menstrual cycle lasting less than three months.

## Figures and Tables

**Table 1:** Correlation analysis (Spearman’s rho index) of the features of the menstrual cycle, anthropometric parameters and characteristics of swimming training in the group of female competitors.

	Menstrual Cycle Disorders	Age of Menarche Appearance	Total of PMS Symptoms Exprienced	Total of PMS Symptoms Alleviated by Swimming	Pain Felt during Menstrual Bleeding according to the Criterion: No Pain. Pain Felt at the Beginning of Bleeding. Pain Felt throughout the Bleeding	Pain Felt during Menstrual Bleeding Assessed Using the Numeric Rating Scale (0–10)	Influence of Swimming Training Undertaken during Menstrual Bleeding on the Degree of Pain Related to Menstruation
Age [years]	−0.02	0.12	−0.12	−0.31 *	0.06	0.23	0.16
Height [cm]	−0.04	0.08	0.05	0.07	0.06	0.09	0.19
Body weight [kg]	−0.27 *	−0.04	0.05	−0.06	−0.13	0.09	0.07
BMI [kg/m^2^]	−0.32 **	−0.16	0.09	−0.09	−0.24	0.10	−0.02
Years of swimming training	−0.16	−0.08	−0.05	−0.26 *	0.09	0.13	0.24
Number of trainings per week	−0.16	0.17	−0.06	0.02	0.21	−0.07	−0.16
The duration of a single swimming training session	0.06	0.08	0.10	0.01	0.10	−0.02	0.16
Number of swimming hours per week	0.13	−0.05	0.00	−0.02	0.00	−0.02	0.17
The number of kilometers swam during one training session	0.11	0.07	0.11	0.03	0.00	−0.03	0.06
Subjective assessment of the experienced effort according to the Borg scale	0.06	−0.01	0.13	0.11	0.16	0.14	0.19

* p < 0.05; ** *p <* 0.01.

**Table 2:** The dominant swimming style undertaken by the athletes and its influence on disorders of the menstrual cycle.

*chi*^2^(6) = 15.22; *p* = 0.019		The Dominant Swimming Style							
		Freestyle		Backstroke		Breaststroke		In Total	
		N	%	N	%	N	%	N	%
Has menstrual period ever been missed for a long time after a period of regular bleeding?	Less than 3 months	12	34.29	7	70.00	4	21.05	23	35.94
	Between 3 and 6 months	1	2.86	0	0.00	5	26.32	6	9.38
	More than 6 months	9	25.71	2	20.00	3	15.79	14	21.88
	There was no such situation	13	37.14	1	10.00	7	36.84	21	32.81
	In total	35	100.00	10	100.00	19	100.00	64	100.00

**Table 3:** The dominant swimming style undertaken by the athletes and its influence on the characteristics of menstrual bleeding.

		The Dominant Swimming Style							
		Freestyle		Backstroke		Breaststroke		In Total	
*chi*^2^(2) = 1.23; *p* = 0.542									
		N	%	N	%	N	%	N	%
Menstruation painfulness	Painful at the beginning	28	82.35	9	90.00	14	73.68	51	80.95
	Painful all the time	6	17.65	1	10.00	5	26.32	12	19.05
	In total	34	100.00	10	100.00	19	100.00	63	100.00
*chi*^2^(6) = 3.77; *p* = 0.708									
		N	%	N	%	N	%	N	%
Does swimming training during menstruation affect the degree of pain feeling?	It reduces pain	19	54.29	3	30.00	11	57.89	33	51.56
	It increases pain	5	14.29	1	10.00	1	5.26	7	10.94
	No influence is observed	9	25.71	5	50.00	6	31.58	20	31.25
	Does not train during menstruation	2	5.71	1	10.00	1	5.26	4	6.25
	In total	35	100.00	10	100.00	19	100.00	64	100.00
*chi*^2^(2) = 0.28; *p* = 0.869									
		N	%	N	%	N	%	N	%
Bleeding is less heavy	No	31	88.57	9	90.00	16	84.21	56	87.50
	Yes	4	11.43	1	10.00	3	15.79	8	12.50
	In total	35	100.00	10	100.00	19	100.00	64	100.00
*chi*^2^(2) = 3.08; *p* = 0.215									
		N	%	N	%	N	%	N	%
Bleeding is more heavy	No	24	68.57	8	80.00	17	89.47	49	76.56
	Yes	11	31.43	2	20.00	2	10.53	15	23.44
	In total	35	100.00	10	100.00	19	100.00	64	100.00
*chi*^2^(2) = 0.07; *p* = 0.964									
		N	%	N	%	N	%	N	%
Bleeding lasts less than 3 days	No	26	74.29	7	70.00	14	73.68	47	73.44
	Yes	9	25.71	3	30.00	5	26.32	17	26.56
	In total	35	100.00	10	100.00	19	100.00	64	100.00
*chi*^2^(2) = 0.71; *p* = 0.700									
		N	%	N	%	N	%	N	%
Amenorrhea	No	30	85.71	9	90.00	15	78.95	54	84.38
	Yes	5	14.29	1	10.00	4	21.05	10	15.63
	In total	35	100.00	10	100.00	19	100.00	64	100.00

**Table 4:** Correlation analysis (Spearman’s rho index) of symptoms characterizing menstrual bleeding and the frequency of injuries appearance as well as anthropometric parameters and characteristics of swimming training in the group of female athletes.

	Less Profuse Bleeding	More Profuse Bleeding	Bleeding Lasts Less than 3 Days	Bleeding Lasts More than 3 Days	Amenorrhea (Monthly Cycle Stopped)	Injuries Incidence
Age[years]	−0.10	0.17	0.00	−0.25 *	−0.06	0.12
Height [cm]	0.01	0.04	−0.07	0.25 *	0.10	0.29 *
Body weight [kg]	−0.21	0.06	−0.03	0.23	0.11	0.28 *
BMI [kg/m^2^]	−0.25 *	0.08	0.02	0.14	0.11	0.21
Number of years of swimming training	−0.12	0.32 *	−0.17	−0.27*	0.03	0.31 *
Number of trainings per week	−0.08	−0.12	−0.12	−0.04	−0.12	−0.04
The duration of a single swimming training session	0.11	−0.04	0.14	−0.23	−0.11	0.11
Number of swimming hours per week	0.05	−0.02	0.11	−0.13	−0.02	0.08
The number of kilometers swam during one training session	0.01	0.04	0.27 *	−0.20	−0.04	−0.11
Subjective assessment of the perceived effort according to the Borg scale	0.17	−0.01	0.07	0.10	−0.02	0.04

* *p* < 0.05.

**Table 5:** Multiple logistic regressions analysis of the predictors: age, height, body weight, BMI, years of training, number of trainings per week, duration of a single training, swimming hours, kilometers, perceived effort associated with less profuse bleeding, more profuse bleeding, bleeding lasts less than 3 days, amenorrhea, and injuries incidence.

Dependent	Predictors	B	Wald	*p*	OR	LLCI	UCLI
Less profuse bleeding R^2^_CS_ = 0.24	age	−0.27	1.33	0.248	0.77	0.49	1.20
	height	1.00	1.38	0.241	2.73	0.51	14.56
	body weight	−1.21	1.04	0.307	0.30	0.03	3.04
	BMI	2.89	0.69	0.405	18.06	0.02	16,445.46
	years of training	−0.36	3.10	0.078	0.70	0.47	1.04
	number of trainings per week	0.03	0.00	0.960	1.03	0.30	3.50
	duration of a single training	1.70	2.25	0.134	5.46	0.59	50.22
	swimming hours	4.91	3.11	0.078	136.23	0.58	32,134.01
	kilometers swam during 1 training	−2.39	3.27	0.071	0.09	0.01	1.22
	perceived effort	0.69	2.57	0.109	2.00	0.86	4.68
More profuse bleeding R^2^_CS_ = 0.21	age	0.17	0.64	0.422	1.18	0.79	1.77
	height	−0.02	0.00	0.968	0.98	0.30	3.15
	body weight	0.04	0.00	0.961	1.04	0.22	4.90
	BMI	−0.03	0.00	0.989	0.97	0.01	104.47
	years of training	0.24	4.62	0.032	1.27	1.02	1.58
	number of trainings per week	−1.19	3.39	0.066	0.30	0.08	1.08
	duration of a single training	−1.66	2.93	0.087	0.19	0.03	1.27
	swimming hours	−0.09	0.00	0.951	0.91	0.05	16.22
	kilometers swam during 1 training	0.46	0.42	0.515	1.58	0.40	6.21
	perceived effort	0.08	0.16	0.691	1.08	0.74	1.58
Bleeding lasts less than 3 days R^2^_CS_ = 0.19	age	−0.07	0.17	0.682	0.93	0.67	1.31
	height	1.01	2.58	0.108	2.74	0.80	9.36
	body weight	−1.42	2.86	0.091	0.24	0.05	1.25
	BMI	4.24	2.92	0.087	69.66	0.54	9022.81
	years of training	−0.11	1.12	0.291	0.89	0.72	1.10
	number of trainings per week	−0.17	0.13	0.716	0.84	0.34	2.11
	duration of a single training	0.44	0.29	0.588	1.56	0.31	7.71
	swimming hours	0.20	0.02	0.894	1.22	0.06	24.00
	kilometers swam during 1 training	1.06	2.22	0.136	2.88	0.72	11.61
	perceived effort	−0.15	0.46	0.496	0.86	0.57	1.32
Amenorrhea R^2^_CS_ = 0.05	age	−0.07	0.10	0.749	0.94	0.63	1.40
	height	0.46	0.52	0.472	1.58	0.45	5.54
	body weight	−0.55	0.42	0.515	0.58	0.11	3.02
	BMI	1.66	0.42	0.517	5.24	0.03	789.89
	years of training	0.03	0.12	0.728	1.04	0.85	1.26
	number of trainings per week	−0.88	1.78	0.182	0.42	0.11	1.51
	duration of a single training	−0.30	0.09	0.763	0.74	0.11	5.19
	swimming hours	−0.57	0.14	0.706	0.56	0.03	11.03
	kilometers swam during 1 training	0.06	0.01	0.936	1.06	0.25	4.45
	perceived effort	−0.03	0.02	0.889	0.97	0.66	1.44
Injuries incidence R^2^_CS_ = 0.23	age	0.19	0.50	0.481	1.21	0.71	2.05
	height	−0.24	0.14	0.708	0.79	0.23	2.75
	body weight	0.54	0.42	0.516	1.72	0.33	8.84
	BMI	−1.47	0.34	0.560	0.23	0.00	32.22
	years of training	0.11	0.63	0.427	1.12	0.85	1.48
	number of trainings per week	−0.39	0.42	0.515	0.68	0.21	2.19
	duration of a single training	−0.01	0.00	0.996	0.99	0.12	8.14
	swimming hours	4.08	2.91	0.088	59.02	0.54	6410.08
	kilometers swam during 1 training	−1.75	2.77	0.096	0.17	0.02	1.37
	perceived effort	−0.07	0.08	0.774	0.93	0.57	1.52

B: regression coefficient; Wald: Wald statistic; OR: odds ratio; LLCI, ULCI: lower and upper limits of 95% confidence intervals for the odds ratio. Constants omitted for simplicity.

## Data Availability

The datasets used and/or analyzed during the current study are available from the corresponding author upon reasonable request.
